# Development and evaluation of a new luciferase immunosorbent assay to detect GII.6 norovirus-specific IgG in different domestic and wild animals

**DOI:** 10.3389/fmicb.2023.1213007

**Published:** 2023-07-20

**Authors:** Zhiyan Liang, Minyi Zhang, Yu Wang, Mark Momoh Koroma, Jingrong Yu, Feiyuan Zhou, Duona Jing, Jiaheng Li, Shixing Tang, Qing Chen, Ying-Chun Dai

**Affiliations:** Department of Epidemiology, School of Public Health, Southern Medical University (Guangdong Provincial Key Laboratory of Tropical Disease Research), Guangzhou, Guangdong, China

**Keywords:** GII.6 norovirus, LISA, domestic and wild animals, IgG, detection

## Abstract

Noroviruses (NoVs) are the leading viral pathogens globally causing acute gastroenteritis (AGE) in humans, posing a significant global health threat and economic burden. Recent investigations revealed that human NoVs had been detected in different animals, which raises concerns about whether NoVs are potential zoonotic diseases. This study developed a novel luciferase immunosorbent assay (LISA) to detect GII.6 NoV IgG based on P protein of VP1. The LISA showed high specificity (99.20%) and sensitivity (92.00%) with 4–16 times more sensitivity compared with an ELISA. NoV-LISA was reproducible with human serum regarding the inter- and intra-assay coefficient of variance values. Potential cross-reactivity was also evaluated using mice serum immunized by other antigens, which showed that NoV-LISA could differentiate GII.6 NoV from rotavirus and various genotypes of NoV. Specific GII.6 NoV IgG was widely detected in different domestic and wild animals, including dogs, pigs, bats, rats, and home shrews, with various IgG-positive rates ranging from 2.5 to 74.4%. In conclusion, our newly developed NoV-LISA assay is suitable for NoV-specific IgG detection in humans and animals. The wide distribution of IgG antibodies against human NoV indicates potential zoonotic transmission between humans and animals.

## 1. Introduction

Noroviruses (NoVs) remain the predominant cause of viral AGE globally, accounting for ~18% of all AGE (Van Beek et al., 2018). According to the World Health Organization (WHO), NoVs are responsible for an estimated 690 million cases of diarrhea and ~220,000 deaths annually, causing a significant disease burden worldwide, particularly in developing countries (Pires et al., 2015). While individuals of all ages are susceptible to NoVs, under-five children experience the highest incidence of infections (Lopman et al., 2016), and people over 80 years old have the highest mortality rate (Zhang et al., 2022).

Noroviruses are a member of the *Caliciviridae* family with a single-strand positive-sense RNA genome of ~7.7 kb in length (Deval et al., 2017). Except for murine NoV, most of the NoV genome is divided into three open reading frames (ORFs) (Chhabra et al., 2019). ORF1 encodes non-structural proteins, including RNA-dependent RNA polymerase (RdRp), while ORF3 encodes for the minor capsid proteins (VP2). ORF2 encodes the major capsid protein (VP1), which is the main antigenic determinant and is divided into the protruding (P) domain and the shell (S) domain (Bertolotti-Ciarlet et al., 2003). Based on amino acid sequences encoding the VP1 protein and RdRp, there are at least 10 genogroups in the NoV genus and over 40 distinct genotypes (Vinjé, 2015). Of the 10 genogroups, GI, GII, and GIV are known to infect humans, with GI and GII being the most predominant cause of NoV infections (Barclay et al., 2021).

Emerging and re-emerging zoonotic diseases continue to be one of the greatest threats to global public health, potentially affecting millions of individuals with devastating consequences (Otte and Pica-Ciamarra, 2021). There have been reports of the spread of zoonotic diseases between humans and other mammals, including inter-species transmission involving other genera of the *Caliciviridae* family (Rahman et al., 2020). NoV, being a member of the *Calicivirdae*, has been considered a potential zoonotic pathogen and infects a wide range of hosts, including pets, livestock, and wild animals. Human NoVs (HuNoVs) have been detected in animals, while antibodies against animal NoVs were also detected in human sera, suggesting a possible risk of zoonotic transmission (Villabruna et al., 2020). The detection of HuNoVs and human-like genotypes in stool samples of farm animals and wild mammals have drawn attention to the potential involvement of animals as a reservoir for various NoV strains. A few serological studies of animal NoVs were reported. For instance, a survey found a variable prevalence of antibodies against GVI.2 NoV in dogs, ranging from 0 to 60% (Mesquita et al., 2014). Another survey found the prevalence of antibodies against GII.11 at 71 and 36% of pigs in the United States and Japan (Farkas et al., 2005). Fewer studies have been conducted to detect HuNoVs in animals. The seroprevalence of HuNoVs in dogs in the United Kingdom was reported to be 13% (Caddy et al., 2013). Serological evidence of NoV infection in domestic animals was limited, and no data were available in wild animals.

In this study, we developed and evaluated a novel luciferase immunosorbent assay (LISA) for detecting specific IgG antibodies of NoVs in humans and animals. Additionally, anti-GII.6 NoV IgG in domestic animals (dogs and pigs) and wild animals (bats, rats, and home shrews) was tested to determine detection rates of anti-HuNoVs IgG, which provided new insights into potential zoonotic transmission on HuNoVs.

## 2. Materials and methods

### 2.1. Samples

To investigate the presence of NoV antibodies in animal serum and establish serological evidence regarding the zoonotic potential of NoV, we collected serum samples from both humans and animals. The sample collection process followed previously published human and animal serum sample methods.

Human serum samples were obtained from the Jidong community cohort study, as published (Wang et al., 2016). In addition, animal serum samples were collected from a diverse range of domestic and wild animals (Table 1). Pig serum samples (*n* = 82) were collected in Guangzhou between January and August 2013, while dog serum samples (*n* = 72) were collected in Zhanjiang. Rat serum samples (*n* = 128; *Rattus norvegicus*: *n* = 127, *Rattus tanezumi*/*Rattus rattus*: *n* = 1) and house shrew serum samples (*n* = 93) were collected between June 2015 and May 2016 in areas where human habitation was present in Guangzhou. Bat serum samples (*n* = 43; *Scotophilus kuhlii*: *n* = 32, *Rousettus leschenaultii*: *n* = 11) were collected between July 2013 and 2016 in Haikou and Zhanjiang (Yang et al., 2004; Zhou et al., 2008; Jiang et al., 2015; Ge et al., 2019). Notably, all animals appeared healthy and exhibited no signs of disease during the blood collection process. Additionally, serum samples were obtained from rats, house shrews, and bats by administering diethyl ether as an anesthetic before the extraction of cardiac blood. Similarly, sterile techniques were used to collect blood samples from dogs and pigs. After collection, the serum samples were isolated via centrifugation and subsequently stored at a temperature of −80°C until further analysis.

**Table 1:** Summary of serum samples of animals.

**Genus**	**Number of serum samples**	**Location**	**Species**	**Year**
Pig	82	Guangzhou, Guangdong	Domestic swine	January–August 2013
Dog	72	Zhanjiang, Guangdong	Chinese rural dog	-
Rat	128	Guangzhou, Guangdong	*Rattus norvegicus* (*n* = 127) *Rattus tanezumi*/*Rattus rattus* (*n* = 1)	June 2015–May 2016
Home shrew	93	Guangzhou, Guangdong	*Suncus murinus*	June 2015–May 2016
Bat	43	Zhanjiang, Guangdong Haikou, Hainan	*Scotophilus kuhlii* (*n* = 32) *Rousettus leschenaultii* (*n* = 11)	July 2013–November 2016

In addition, we adhered to the established methodologies for mice to produce serum samples that were either hyper-immune or non-immune. Hyper-immune serum samples targeting rotavirus and NoV, along with non-immune sera, were generated using the described techniques (Zhang et al., 2016; Dai et al., 2017; Xie et al., 2020).

### 2.2. Construction of recombination expression plasmid pNLF-P

**Figure 1:**
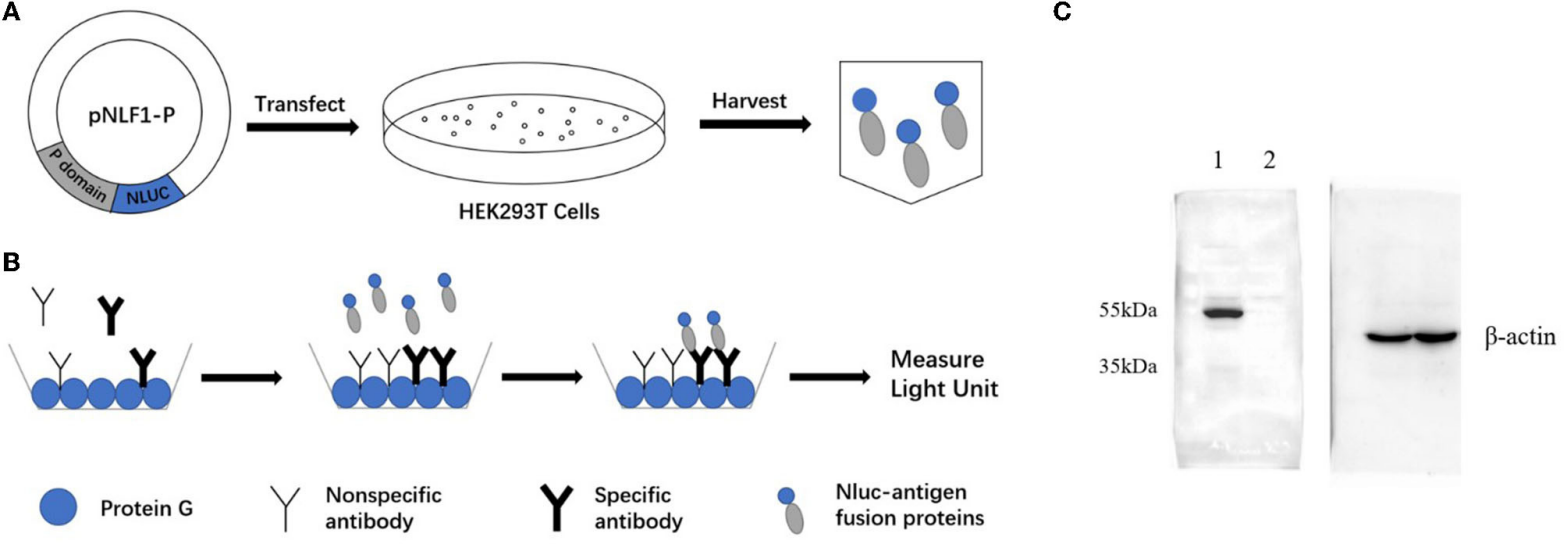
Schematic diagrams of LISA **(A, B)** and Western blot analysis of the NLuc-antigen fusion protein **(C)**. **(A)** The recombinant plasmid pNLF1-P was constructed and transfected to the HEK293T cells to generate the luciferase-antigen lysates. **(B)** Microtiter plates coated with protein G were incubated with serum samples. Luciferase-antigen lysates were then added for IgG antibody capture determined by adding the nano-luciferase substrate. **(C)** Western blot identification of the NLu-P fusion protein. Beta-actin was used as an internal control; lane 1, pNLF1-P; lane 2, pNLF1-N.

The recombination expression plasmid pNLF-P (Figure 1A) was constructed using the luciferase expression vector pNLF1-N (Promega, USA). Fragments of the norovirus GII.6 P domain (NCBI Accession no. MT861044) were amplified from the plasmid pGEX-4T-GII.6P through PCR, employing the following primers: 5′ CCGGAATTCTCAAAGACTAAGCCCTTTAC−3′ and 5′- TAAAGCGGCCGCTTAGCAAAAGCAATCGC−3′. The genome structure of NoV can be found in Supplementary Figure 1. The PCR was carried out under the following conditions: pre-denaturation at 95°C for 5 min, followed by 35 cycles of denaturation at 95°C for 30 s, annealing at 55°C for 30 s, extension at 72°C for 1 min, and a final extension at 72°C for 10 min. Subsequently, the PCR products were inserted into the EcoRI-NotI-cut pNLF1-N vector after double digestion with EcoRI and NotI restriction enzymes (Takara Biotechnology Co., Ltd, China). The resulting recombinant plasmids were verified through DNA sequencing to confirm the absence of PCR-induced errors and were used for cell transfection.

### 2.3. Expression of NoV P antigens fused with nano-luciferase

HEK293T cells were seeded into 100-mm^2^ dishes in 5% CO_2_ at 37°C. After 24 h of cell plating, the cell density reached 70–90%, and the cells were transfected with 5 μg of recombinant plasmid DNA using Lipofectamine 3000 (Invitrogen, USA) according to the manufacturer's protocols. Briefly, 5 μg of recombinant plasmid DNA was diluted with 240μl of Opti-MEN™ reduced serum medium (Gibco, USA) and mixed with 10μl of P3000™ reagent (Invitrogen, USA) followed by incubation at room temperature for 10 min. Subsequently, the DNA complex was added to the cells and cultured for 48 h. Afterward, the cells were washed twice with cold 0.01M phosphate-buffered saline and trypsinized.

The cells were then washed once with PBS and lysed on ice for 30 min using lysis buffer. The supernatants were collected by centrifugation at 12,000 rpm for 4 min at 4°C and stored at −80°C.

To measure luciferase activity in the crude cell extracts, an equal volume of Nano-Glo Luciferase assay reagent (Promega, USA) was added, and the measurement was performed using a Tecan Infinite M200 PRO microplate luminometer.

### 2.4. SDS-PAGE and Western blot analysis

The expression of luciferase-tagged P protein was confirmed using Western blot (WB) analysis. To ensure equal loading of the target protein and negative control, the protein concentrations of the lysates were determined using BCA protein assays. Subsequently, appropriate amounts of the lysate were separated on a 10% sodium dodecyl sulfate–polyacrylamide gel (SDS-PAGE) after the samples were heated at 100°C for 10 min (Beyotime Co., China). For the WB analysis, the proteins were electro-transferred onto a 0.22-μM nitrocellulose membrane at 15 V for 45 min. The membrane was blocked overnight at 4°C with 5% non-fat dry milk (NFDM) in PBS. As primary antibodies, a murine monoclonal antibody against GII.6 NoV (dilution, 1:2000), generated by our group (Zhang et al., 2020), or a mouse anti-beta-actin monoclonal antibody (dilution, 1:10000), were used accordingly. The secondary antibody used was a goat HRP-conjugated anti-mouse IgG antibody (Abcam Co., China) diluted 1:4000 in 3% NFDM.

Peroxidase activity was detected using a Western blotting detection reagent (Amersham ECL Prime; GE Healthcare, Tokyo, Japan) and a luminometer analyzer (LAS-4000; FujiFilm, Tokyo, Japan).

### 2.5. ELISA

The in-house ELISA for detecting NoV-specific IgG antibodies was performed as described previously (Xie et al., 2020; Zhang et al., 2020). In brief, the plates were coated with 0.5 μg/mL of diluted GII.6 VP1 P protein. After blocking with 5% non-fat milk in PBS, serum samples of humans or rats were added. The IgG antibodies were then detected by horseradish peroxidase (HRP)-conjugated goat anti-human IgG or HRP-conjugated goat anti-rat IgG diluted at a concentration of 1:3000. The cutoff value was set at OD_450_ = 0.2, a value of the mean of the background/blank wells adding a triple standard deviation (Xie et al., 2020; Zhang et al., 2020). To keep the results consistent and reproducible, positive and negative controls were involved in each plate, and all experiments were performed at least in duplicate.

### 2.6. Development of P protein-based LISA for IgG detection of GII.6 NoV

Microtiter plates (Greiner Bio-One Co., China) were coated with protein G (5 μg/mL, 50 μL/well; Genscript Co., China) in PBS overnight (14h) at 4°C (Figure 1B). After washing with PBS containing 0.05% Tween-20 (PBS-T) and blocking with 5% non-fat milk in PBS for 2 h at 37°C, 50 μL of human serum samples at a dilution of 1: 500 or animal samples at a dilution of 1: 100 were added and then incubated for 1 h at 37°C. After washing three times, 50 μL of NLuc-antigen lysates of 2% non-fat milk in PBS (equal to 2 × 10^5^ light units) were added and incubated at 37°C for 30 min. After washing, 50 μL of the Nano-Glo Luciferase substrate was added to each well to determine the LU according to the manufacturer's protocol. The average LU for the negative samples was calculated, and the cutoff value was set to twice the negative controls confirmed by ELISA, as recommended in previous publications (Wang et al., 2019, 2020). All experiments were performed at least in triplicate.

### 2.7. Comparison of the limit of detection (LOD) between ELISA and LISA, repeatability, and cross-reactivity of NoV-LISA

The limit of detection (LOD) refers to the minimum concentration or quantity of the target substance that can be reliably detected using a specific analytical method. To assess the LOD of LISA and ELISA, five human serum samples were selected based on their positive anti-GII.6 NoV IgG status, with high values of light units (LU) and OD_450_. These samples were serially diluted, allowing for the evaluation of the LOD of both LISA and ELISA.

For the evaluation of LISA stability, nine human samples were chosen, comprising three samples from each of the following categories: high grade (OD_450_ > 1.3), medium grade (0.8 ≤ OD_450_ ≤ 1.3), and low grade (OD_450_ < 0.8). Each sample was tested in triplicate across three different plates, ensuring repeatability and providing insights into the stability of LISA.

To determine the cross-reactivity of NoV-LISA, in-house hyper-immune serum samples generated in mice against VP8^*^ proteins of rotavirus and VP1 P proteins of NoV, as well as non-immune sera, were employed. These hyper-immune and non-immune serum samples were produced following the earlier methodologies described (Zhang et al., 2016; Dai et al., 2017; Xie et al., 2020). The cross-reactivity of NoV-LISA was evaluated using these serum samples.

### 2.8. Statistical analysis

All the data of LU and OD_450_ were presented as the average of triplicate or duplicate tests. The efficacy of the NoV-LISA was determined by plotting the area under the receiver operating characteristic curve (AUC). The sensitivity, specificity, positive likelihood ratio (+LR), and negative likelihood ratio (-LR) were calculated using MEDCALC online (www.medcalc.org). The inter- and intra-assay coefficient of variance values (CV) were determined by dividing the standard deviation by the mean (%). Group comparisons were performed using analysis of variance (ANOVA). The correlations were analyzed using Pearson's correlation coefficient. The data were analyzed using SPSS 26.0 statistical software (SPSS Inc., Chicago, USA) and GraphPad Prism 8.0 (GraphPad Software, California, USA). A *P*-value of < 0.05 is defined as statistical significance.

## 3. Results

### 3.1. Comparison of the novel NoV-LISA with ELISA for the detection of anti-GII.6 NoV IgG

**Figure 2:**
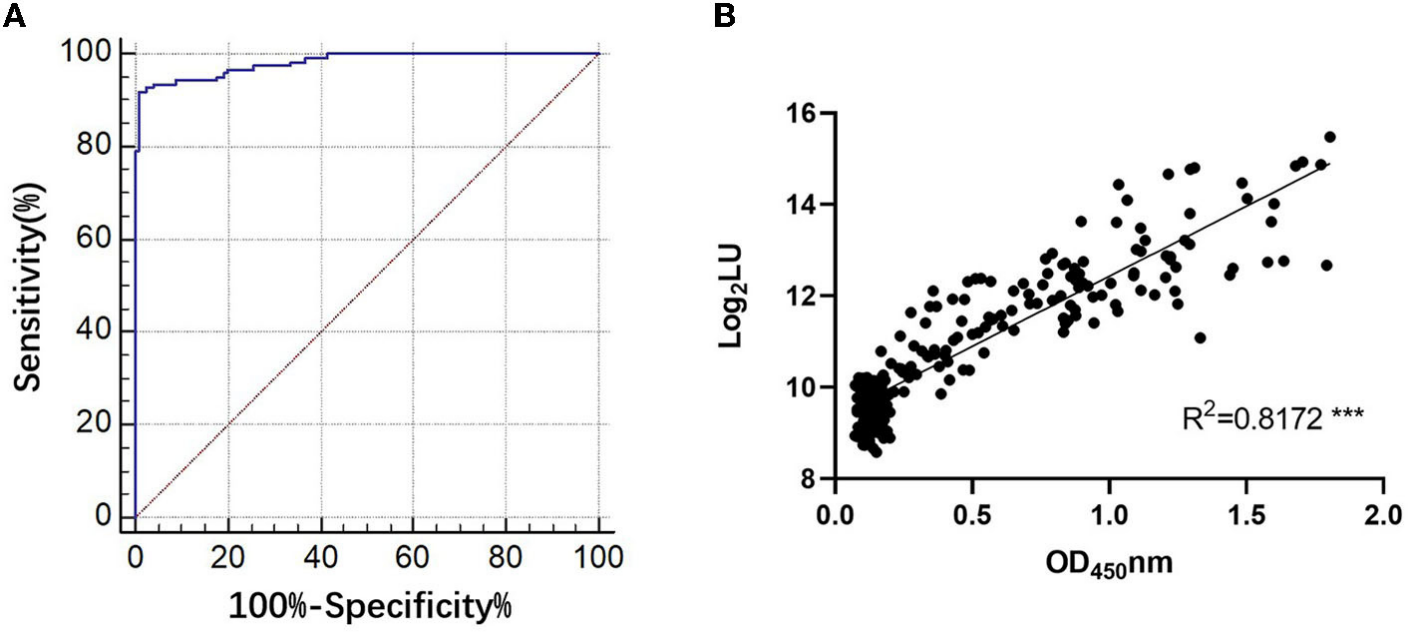
Evaluation of the performance of NoV-LISA. **(A)** The sensitivity and specificity of LISA and the positive and negative likelihood ratios were evaluated using the area under the curve in the ROC analysis. **(B)** The correlations between LISA (log_2_ LU) and ELISA (ratio) for detecting IgG antibodies. A total of 250 human serum samples were selected for evaluating the correlation between ELISA and NoV-LISA. The ratio of the ELISA results was plotted against the log_2_ LU of the LISA.

The NLuc-P fusion protein was successfully expressed using WB analysis (Figure 1C). Human serum samples (*n* = 250) with half positive and half negative to anti-GII.6 NoV IgG defined using ELISA were selected for the evaluation of NoV-LISA. LU at 1,600 was set as the cutoff value, twice the average value of negative samples at 800 LU. The sensitivity and specificity of LISA were 92.00 and 99.20%, respectively, as evaluated in the ROC analysis. The area under the curve (AUC) was calculated to be 0.982. The newly developed LISA exhibited high performance with a positive likelihood ratio (+LR) at 115.0 and a negative likelihood ratio (-LR) of 0.081 (Figure 2A; Table 2). A strong correlation between LISA and ELISA was also observed in scatter plots with Pearson's correlation coefficients at 0.82 (*P* < 0.01, Figure 2B).

**Table 2:** Evaluation of the results of NoV-LISA.

**Characteristics**	**Results (95% CI)**	**High-performance range**
Sensitivity	99.20% (95.6–100.0%)	–
Specificity	92.00% (85.8–96.1%)	–
Area under the ROC curve (AUC-ROC)	0.982 (0.957–0.995)	0.9–1
Positive likelihood ratio (+LR)	115.00 (63.33–208.90)	≥10
Negative likelihood ratio (-LR)	0.081 (0.04–0.1)	≤ 0.1

**Figure 3:**
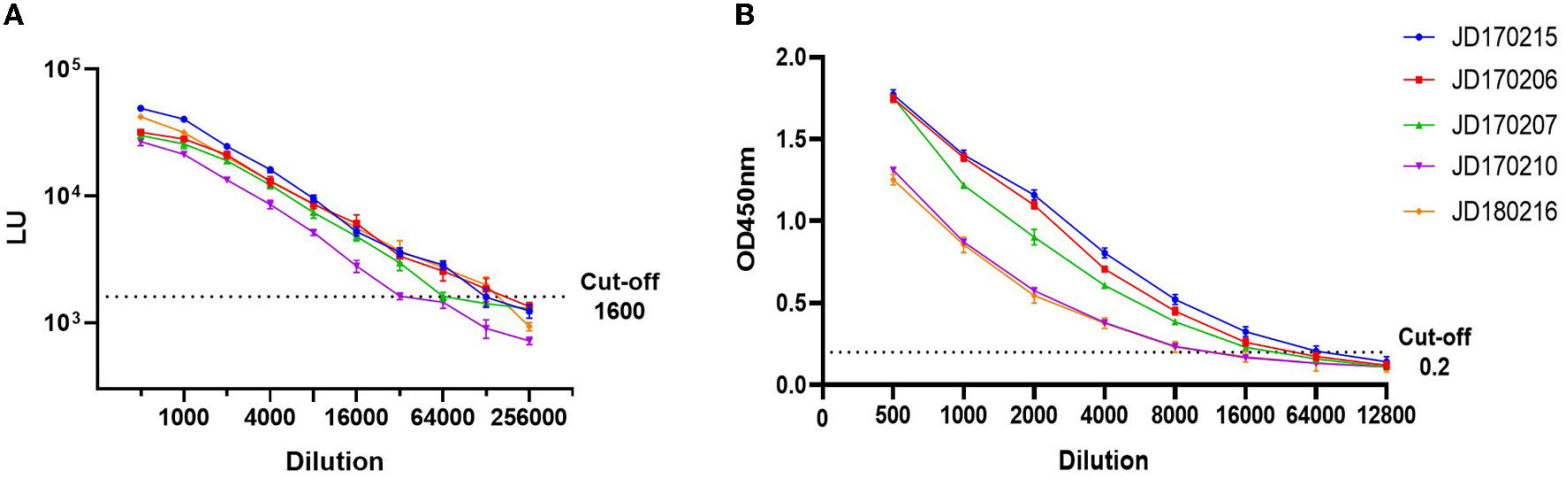
Comparison of the limit of detection (LOD) between LISA **(A)** and ELISA **(B)**. Five serum samples positive for anti-GII.6 NoV IgG, shown by both ELISA and LISA, were selected and serially diluted. The dotted line indicates the cutoff value. Serum samples were represented using the ID codes in the Jidong community cohort.

To compare LOD, five positive serum samples that previously tested positive for anti-GII.6 NoV IgG using both LISA and ELISA methods were used. The LOD of LISA was observed at a serum dilution of 1:64,000–1:128,000 while that of ELISA ranged from 1:8,000 to 1:32,000. The results indicate that the LOD of NoV-LISA was 4- to 16-fold higher than that of the ELISA (Figure 3).

**Figure 4:**
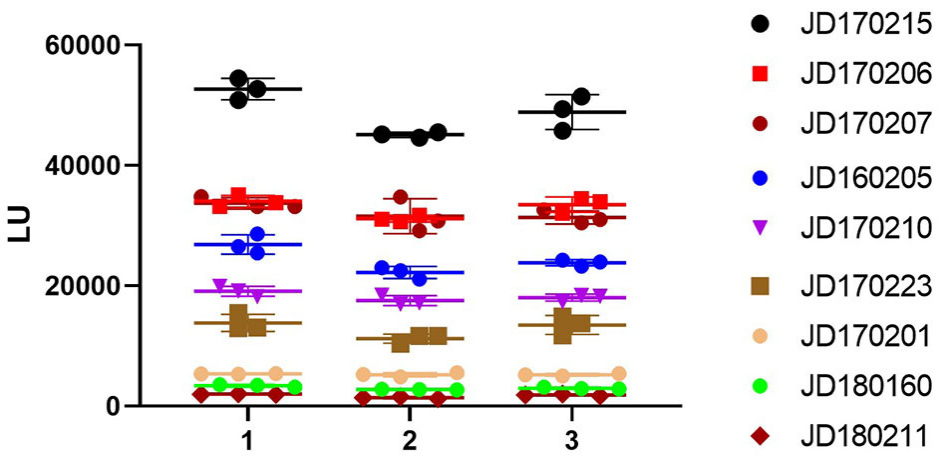
Repeatability of NoV-LISA. Nine positive samples for anti-GII.6 NoV IgG by ELISA were selected from three grades (3 samples of each grade) according to the OD_450_ detected using ELISA, including high (OD_450_ > 1.3), medium (0.8 ≤ OD_450_ ≤ 1.3), and low (OD_450_ < 0.8). Each sample was tested in triplicate on three different plates. The X-axis indicates the different plates used, and serum samples are represented using the ID codes in the Jidong community cohort.

According to the OD_450_ of serum antibody detected using ELISA, human serum samples were divided into three grades: high (OD_450_ > 1.3), medium (0.8 ≤ OD_450_ ≤ 1.3), and low (OD_450_ < 0.8). We selected three samples from each grade (Figure 4). The intra-assay coefficient of variance (CV) of the three grades was found to be 3.1, 4.9, and 4.3%, while the inter-assay CV was found to be 5.8, 8.6, and 9.7%, respectively. Both intra- and inter-assay CV values were less than 15% in each group, demonstrating high reproducibility in each grade.

### 3.2. The cross-reactivity of NoV-LISA

**Figure 5:**
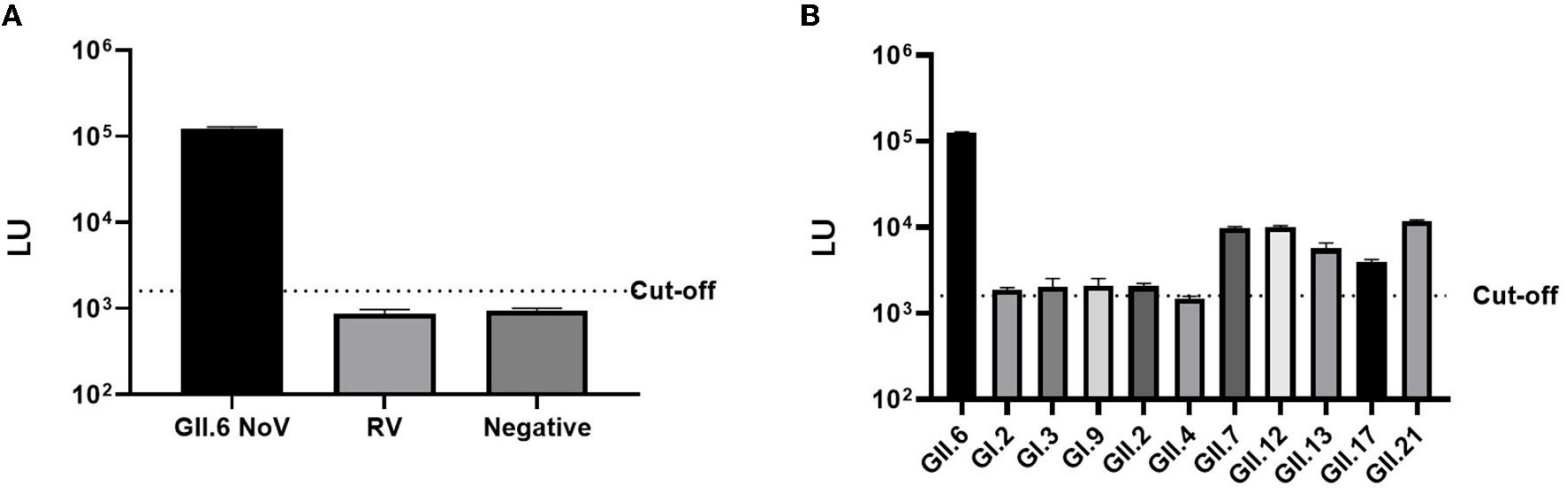
Cross-reactivity of NoV-LISA. The hyper-immune samples against rotavirus **(A)** and different NoV genotypes or non-immune mice serum samples **(B)** were used. The dotted line indicates the cutoff value.

The LU of mice serum immunized with VP1 P protein of GII.6 NoV (>10^5^ LU) was significantly higher than that of the control group immunized with rotavirus (< 10^3^ LU), demonstrating the high specificity of LISA (Figure 5). Although there was a degree of cross-reactivity with some NoV strains (10^3^-10^4^ LU), the LU of the mice serum against GII.6 NoV was significantly higher than sera against other NoV genotypes, especially the serum of GI NoV-immunized mice (10^3^ LU). This finding suggests that NoV-LISA can differentiate GII.6 NoV from other genotypes (Figure 5).

### 3.3. A variety of IgG-positive rates of GII.6 NoV observed in serum samples from domestic and wild animals

**Figure 6:**
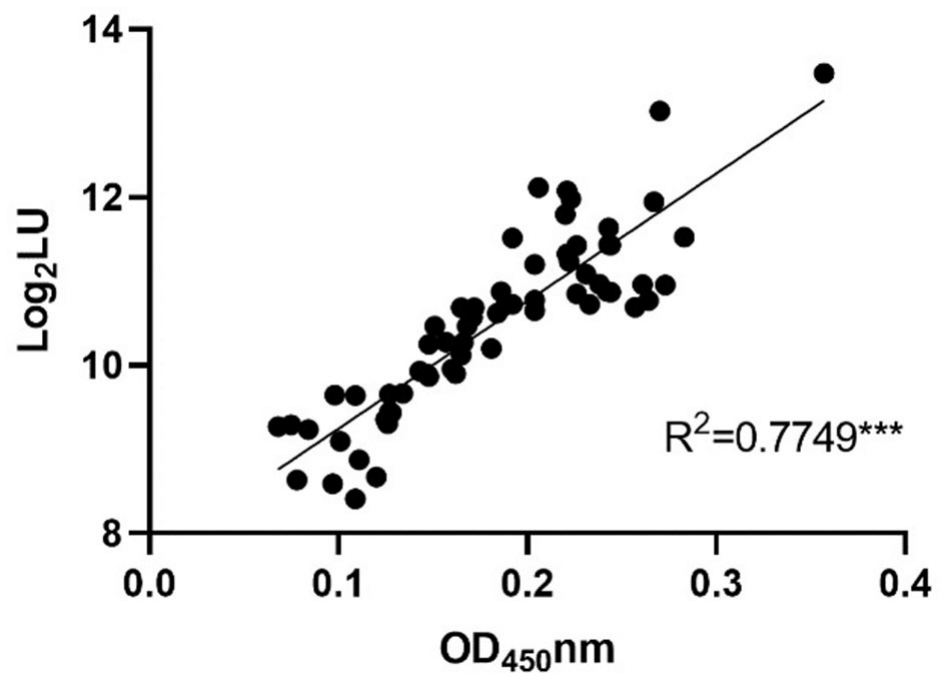
The correlations between LISA (log_2_ LU) and ELISA (ratio) for detecting IgG antibodies in rat sera. A total of 64 serum samples of rats were randomly selected and tested in parallel using both ELISA and LISA. The ratio of the ELISA results was plotted against the log_2_ LU of the LISA.

To further confirm the feasibility of NoV-LISA in animals, a total of 64 serum samples of rats were randomly selected and tested in parallel using both ELISA and LISA (Figure 6). A strong correlation between LISA and ELISA was observed in scatter plots with Pearson's correlation coefficients of 0.7749 (*P* < 0.001). Compared with ELISA, the detection results of all positive samples by LISA in rats were consistent, with a sensitivity of 100% (27/27) an specificity of 83.8% (31/37).

**Figure 7:**
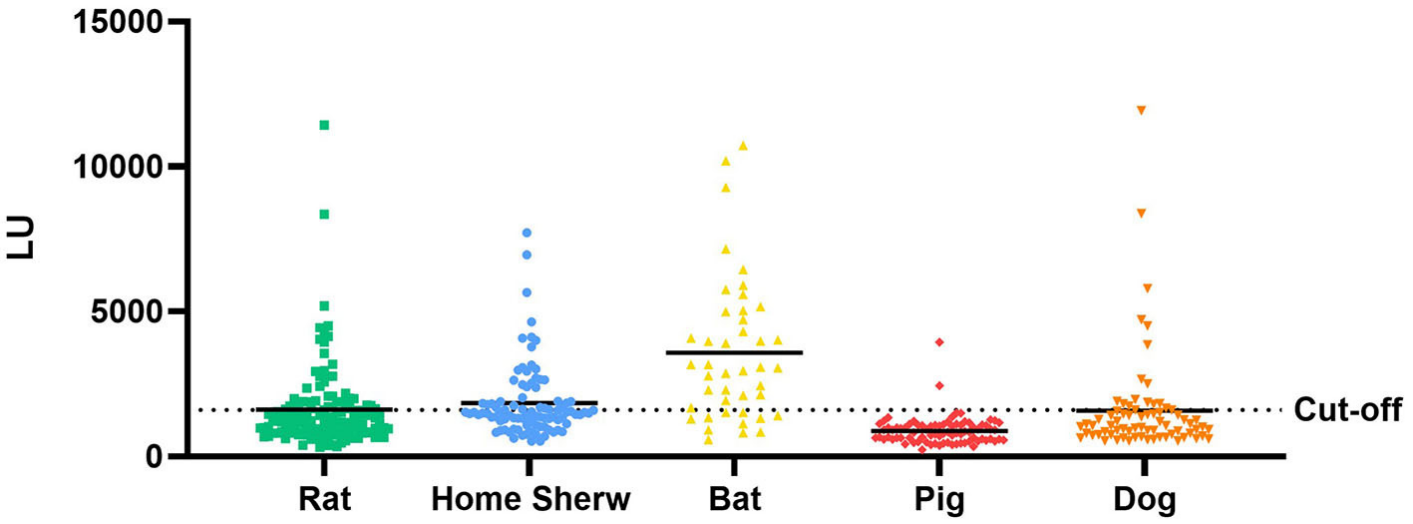
Test results of serum samples from domestic and wild animals using NoV-LISA. Anti-GII.6 NoV IgG was detected in domestic animals (dogs and pigs) and wild animals (rats, shrews, and bats) using NoV-LISA. The dotted line indicates the cutoff value for LISA. The solid line indicates the average LU.

Anti-GII.6 NoVs IgG was detected in the samples of both domestic and wild animals tested; nevertheless, their positive rates spanned a large range (Figure 7). The IgG-seropositive rates of anti-GII.6 NoV in domestic animals were as follows: 23.6% (17/72) in dogs, 2.5% in pigs (2/82), and those in wild animals, such as rats and home shrews, were 32.8% (42/128) and 38.7% (36/93), respectively. However, compared with domestic and other wild species, bats showed the highest rate, up to 74.4% (32/43). *Scotophilus kuhlii* and *Rousettus leschenaultii* were the two bat species from which samples were collected, and they exhibited statistically significant differences in the IgG-positive rates at 93.75% (30/32) and 18.18% (2/11), respectively (*P* < 0.001).

## 4. Discussion

The detection of NoV in humans and animals has led to its classification as a potential zoonotic virus, raising concerns about a possible risk of animal reservoirs for human infections and new modes of transmission (Villabruna et al., 2019). Most of the initial understanding of animals serving as reservoirs for HuNoVs stems from domestic animals (Scipioni et al., 2008). NoV VLPs have been shown to attach to the intestinal tissues of animals, exhibiting potential susceptibilities to different animal species (Villabruna et al., 2021). However, low virus genomes in the RNA detection of NoV can be a result of the ingestion of NoV-contaminated food or other stuff and does not necessarily constitute a true infection (Jothikumar et al., 2005). A specific immune response can more clearly demonstrate whether animals can act as hosts for HuNoVs.

Here, we demonstrated a novel LISA useful for rapidly and sensitively detecting anti-HuNoV IgG in humans and animals. Our study employed LISA to detect NoV infection based on a GII.6 NoV P domain fragment. Extensive epidemiological data indicated that GII.6 had shown continuous and widespread dissemination over the past 30 years. Meanwhile, GII.6 NoV is relatively stable and has a few clusters, making it a good indicator for detecting NoV infection in both animals and humans (Jin et al., 2020). Our study found that both LISA and ELISA yielded consistent results when analyzing human and rat serum samples. Notably, our results further revealed that NoV-LISA demonstrated a much lower LOD for anti-HuNoV antibodies than ELISA. These findings are in line with previous studies that have demonstrated the sensitivity, specificity, repeatability, and cross-reactivity of LISA as a unique detection assay compared with other detection assays (Oyama et al., 2021). One of the significant advantages of LISA is that it does not require purified antigens, which can be difficult and time-consuming to obtain (Baeshen et al., 2015). At the same time, ELISA requires the recombinant antigen expressed in a prokaryotic or eukaryotic expression system. These methods may be time-consuming, laborious, have low antigens, produce inactive or insoluble proteins, and lack post-translational modifications. Another important feature of LISA is that it eliminates the need for species-specific secondary antibodies, making it particularly useful for detecting antibodies in wild animals (Wang et al., 2020). In addition, compared with ELISA, LISA does not need chromogenic time, and NLuc is more sensitive than conventional HRP. Furthermore, LISA can be used in serological studies of field viruses and provide information about the frequency of a pathogen in a certain host species. These features make LISA an excellent tool for screening host exposure risk, cross-species transmission, and source tracing, especially in situations in which other diagnostic methods may not be feasible.

In this study, we used the NoV-LISA assay to detect anti-GII.6 NoV IgG in serum samples from various animal species. The animal species included domestic animals (pigs and dogs) and wild animals (rats, home shrews, and bats). Our results revealed different IgG-positive rates of GII.6 NoV in each represented animal species, suggesting they had been exposed to or infected with HuNoVs. Consistent with our findings, earlier studies have shown domestic animals such as dogs and pigs to be exposed or infected with HuNoVs, possibly due to their proximity to humans (Summa et al., 2012; Bucardo et al., 2016). The similar rates of IgG positivity in rats and home shrews could be attributed to the fact that both species were collected from the same locations and likely had similar exposure to HuNoVs. These animals may have come into contact with human excrement in sewers, potentially leading to infection with HuNoVs (Wolf et al., 2013).

Interestingly, our findings revealed that wild animals had higher detection rates for anti-GII.6 NoV IgG than domestic animals, with bats exhibiting the highest positive rates. Bats are known to be natural reservoirs for many zoonotic viruses, carrying over 100 different viruses, including most of the common infectious diseases (Van Brussel and Holmes, 2022). Additionally, bats have the potential to host a variety of viral diseases; hence, a database has been specifically established to keep track of bat viruses, including *Caliciviruses* (Wu et al., 2016). While there are notable differences between the NoVs found in bats and HuNoVs, detecting a high IgG-positive rate against GII.6 NoV suggests that bats may be carriers of HuNoVs. In particular, *Scotophilus kuhlii* exhibited much higher GII.6 antibodies than *Rousettus leschenaultii*. The current finding aligns with previous research showing that carnivorous bats tend to carry more causative agents than frugivorous bats (Wu et al., 2016). However, further studies are required to establish the precise mechanism by which HuNoVs are transmitted to bats. This study represents the first known instance of HuNoV antibodies being detected in wild animals, thus highlighting the need for additional research in this area.

## 5. Conclusion

In conclusion, we designed a novel P domain-based LISA for the sensitive detection of HuNoV antibodies across species. LISA offers a plausible alternative to ELISA for confirming NoV detection due to its ease of use, high sensitivity, and rapidity. We observed positive rates of NoV-specific IgG in various domestic and wild animals, with a higher rate in wild animals. This finding suggests the potential for zoonotic transmission of HuNoVs between humans and wild animals.

## Data availability statement

The raw data supporting the conclusions of this article will be made available by the authors, without undue reservation.

## Ethics statement

The studies involving human participants were reviewed and approved by the Ethical Committees of the Staff Hospital of Jidong oilfield of China National Petroleum Corporation. The patients/participants provided their written informed consent to participate in this study. The animal study was reviewed and approved by the Animal Ethics and Welfare Committee of the School of Public Health, Southern Medical University.

## Author contributions

Y-CD designed and managed this study, provided project funding, and rewrote the manuscript. QC provided the animal serum samples and the information. ZL finished the experiments and wrote the first draft. MZ collected the animal samples. YW made contributions to experiments, the data analysis, and revised the manuscript. MK participated in writing and critically revising the manuscript. ST provided technical guidance on the development of NoV-LISA. FZ made contributions to analyzing and interpreting data. JY, DJ, and JL helped to develop NoV-LISA and data analysis. All authors contributed to the article and approved the submitted version.
