# Supplementary figures and images for: Development and evaluation of a new luciferase immunosorbent assay to detect GII.6 norovirus-specific IgG in different domestic and wild animals
Source: Front Microbiol. 2023 Jul 20;14:1213007. doi: 10.3389/fmicb.2023.1213007 (PMC10399220; doi:10.3389/fmicb.2023.1213007)

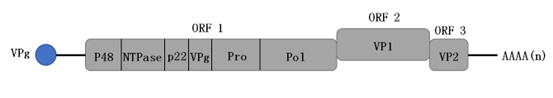

Supplement: Supplementary file 1 [file Image_1.PNG]
